# Hysterectomy for Malignant Diseases of the Uterus

**Published:** 1888-11

**Authors:** Wm. H. Wathen

**Affiliations:** Louisville


### HYSTERECTOMY FOR MALIGNANT DISEASES OF THE UTERUS.

By Dr. Wm. H. Wathen, of Louisville.

[Abstract of a paper read in the American Association of Obstetricians and Gynecologists at Washington, D. C., September 20, 1888, and contributed to Daniel's Texas Medical Journal by the author.]

HYSTERECTOMY for malignant diseases of the uterus was done by Freund in 1878, and is now a recognized operation by nearly all learned gynecologists. He removed the uterus through the abdomen, but the mortality was so great that this means of operating is no longer approved of except in a few cases where it is not possible or practicable to remove it through the vagina. In the abdominal method the mortality was 72 per cent., but it has been reduced by the vaginal method, in the practice of a few expert operators, to 8 or 10 per cent. and in a collection of 311 cases, operated on by Fritsch, Leopold, Alshausen, Schroeder, Staude and Martin, the mortality is only 15:1 per cent. Martin in fifty-five consecutive cases, had a mortality of about 9 per cent. Fritsch in twenty-four consecutive cases had a mortality of about 8 per cent., and Staude operated successfully in sixteen consecutive cases. As the operation is in its infancy, we may expect better results when experience teaches us more exact indications for its performance, and a more perfect technique in the details of the operation and the after management of the case. In the operation for high excission of the cervix the mortality is 7.4 per cent. and by this operation it is impossible to know if we have removed all the tissues involved in the malignant growth, as the disease may have extended high up the endometrium, or in the parenchyma of the body. Nor is it always possible to do so by subjecting the tissue to a microscopical examination, and never possible except the examination be conducted by a microscopist experienced in such matters.

The subsequent results are better after total extirpation than after supravaginal excission. Of course high amputation should not be performed where the endometrium or the parenchyma of body or fundus is primarily or secondarily involved. The disease may extend from the neck to the body and remain there for some time without empoisoning the system, or involving the structures in the immediate proximity of the uterus.

There are, however, a few gynecologists who deny that hysterectomy for cancer is a justifiable operation, among whom is Dr. A. Reeves Jackson, of Chicago, who read a learned paper at the meeting of the Ninth International Medical Congress to show that the operation had destroyed nearly 300 years of human life. This is a fearful exhibit, but statistics are often unreliable, and in many of these cases the disease had doubtless extended beyond the uterus, so that the operation was contradicted ; but experience will guide us more correctly in the future, and results will be better. Kuester, at the meeting of the German Surgical Association, in 1883, reported 778 cases of removal of the cancerous breast, with a mortality of 15.6 per cent., 5 per cent. greater than the general average mortality following total extirpation of the uterus, but is there a distinguished surgeon who would hesitate to amputate the breast where the diagnosis of cancer is made before the disease has manifestly infected the system. If we recognize cancer as primarily local in its origin, and becoming constitutional secondarily by extension, then the correct treatment is to remove, if possible, all the involved tissues if it can be done with a reasonable degree of safety. With few exceptions this can be done in cancer of the uterus by total extirpation only. But if the disease has extended to any structure outside of the uterus, peritoneal, cellular or glandular, or if there is any systemic infection, then the operation is positively contraindicated, and no one but a pseudo-gynecologist, ignorant of the disease, and of the technique of the operation for its removal, would attempt it.

Unfortunately, the popular craze in this country and in Europe to do abdominal surgery, and to record abdominal sections, has included in its grasp vaginal hysterectomy as well, and these operations are now being attempted by men who are not fitted for such work, the result being a fearful mortality, because the operator knows neither when the operation is indicated nor how to perform it.

I have known of some sad examples of this in my own observation. It is positively *criminal* for any one to attempt to extirpate a cancerous uterus, or to do pelvic or abdominal surgery until he learns the anatomy, physiology and pathology of the pelvic and abdominal structures, and knows how to make a correct diagnosis where it is possible to do so; he should also know the general principles and the details of the most approved technique for such operations. It is indeed gratifying to observe that the progressive medical mind has taken cognizance of this abuse and in the future the general practitioner will be more careful in the selection of a proper person to whom he may refer such patients. These operations are among the most heroic and dangerous in surgery, and should not be done except by a person who has devoted much time and careful attention to all the theoretical and pratical details relating to this subject.

The so-called gynecologist who proposes to do work for which he has made no preparation, and for which he has but little aptitude, is controlled more by a desire to advance his personal interests than to do the best thing for his patients, hence he attempts operations that the scientific gynecologist will not perform because he knows they are positively contra-indicated and will result in injury to, and probably death of the woman. It is by no means an easy matter to decide when the operation is indicated, for we are often unable to say that the disease is malignant, or how far it has extended. Unfortunately many practitioners fail to note the early symptoms of cancer and when the woman is referred to the specialist the disease has extended so far that it cannot be removed.

No effort should be made to extirpate the uterus if there is the least evidence of cancerous cachexia and if in a careful physical examination any structure outside of the uterus in the pelvic cavity is at all infected. In every case when the diagnosis is not complete by a complete physical examination of the patient, part of the growth should be excised and carefully examined by a learned and an experienced microscopist, whose opinion will do much to aid us in deciding upon the propriety of an operation. But the microscopist can not always differentiate between a benign growth and cancer. He knows that diseased cervices are more liable to cancer than sound ones, but can scarcely distinguish between the "heterologous glandular development invading new tissues with reduplication and proliferation of epithelium external to the contiguous basement membrane of the glands, and a cancerous degeneration, where the cells, breaking through into the lumin of the glands, grow inward, forming solid plugs or process instead of hollow tubes or acini." The transition of glandular erosion into cancer is often so insiduous that it is nearly impossible to decide just when the change occurs. Nor is it positively shown that cancer always originates in the new-formed glands; the cervix may be infiltrated as a "carcino-sarcoma, an aggregation of small cells, lying in masses, more or less completely separated by partitions of connective tissue," with no direct connection with the epithelium or the glands. Probably the following conclusions of Fuerst are about as reliable as any we have seen, viz.:

1. "Adenoma uteri simplex, or simple glandular hyperplasia, in which the uterine glands are increased in number and enlarged, but still preserve their typical character in structure and as to their epithelial covering—in which also, the interstitial tissue is compressed, though there is no evidence of extraordinary cell proliferation—must be regarded as benign. It is not advisable, however, to treat this condition by curettement, application of caustic substances, and similar means, but by excision, for the new growth tends to malignant degeneration."

2. "Adenoma uteri-suspectum, or destructive glandular hyperplasia, which, whether it is localized or diffuse, shows a new formation of atypical gland tubes, together with more or fewer normal glands imbedded in connective tissue, which is rich in cells—which also shows an increase in the gland epithelium and a tendency to proliferation in its lower portions—must be regarded as a suspicious condition, and inclined to malignancy, even in cases where no solid processes are demonstrable. In such cases excision will not suffice, but the uterus should be extirpated as soon as possible, the prognosis with reference to radical cure being good."

3. "Adeno carcinoma uteri, in which the typical gland formations have almost entirely disappeared, and in which we find epithelial infiltration and cancer processes in a foundation which is rich in cells, is absolutely malignant in character. Even if the uterus is extirpated the prognosis as to radical cure is not good, for the chances of being able to extend the section into healthy tissue are poor, hence the treatment can only be pallative for the greater number of cases.

4. "The clinical symptoms in connection with cervix adenoma, are of less value in determining as to operative procedures than the result of the pathologico-anatomical examination. As early an examination as possible of an excised portion of ihe mucous membrane is therefore imperative."

Asepsis, or perfect surgical cleanliness, should be enforced in every detail of the operation. The operation should not be performed until the bowels are well moved and the woman has washed her entire person in a bath of hot water and pure soap. Two gallons of hot water should be injected into the vagina just before the operation.

Weak solutions of disinfectants may be used, but I doubt their efficacy. The woman should be placed on her back, and when under an influence of an anæsthetic, the uterus should be drawn through the vulva and separated with the scissors or knife from its vaginal attachment, being careful not to wound the bladder or to cut the uterine artery. After separating the uterus from its anterior and posterior attachments, carefully avoiding the bladder, uterus or rectum, long-catch forceps, such as I have devised, or such as are used by other operators, should be fastened on each of the broad ligaments, so as to cut off the blood supply to the uterus. The uterus may then be separated from the broad ligaments with the scissors or knife. This may be done without retroverting or anteverting it. No sutures are usually needed. If there is any considerable hemorrhage from the cut edges of the vagina, it can be readily controlled by applying small catch-forceps, and, if necessary, leaving them in position. If it is desirable to bring together the peritoneal edges of the vaginal opening, this may be done by holding the anterior and posterior edges in apposition by catch-forceps. The smaller forceps may be left in position for twenty-four hours, and the larger ones, including the broad ligaments, may be removed in forty-eight hours. By this means the uterus may be extirpated in less than half the time consumed in operating after the fashion of Shroeder and Martin, and the loss of blood should be too insignificant to be an important factor. But no one should attempt this operation until he is prepared and equipped to meet any emergency that may possibly arise.

